# From Electrolyte to Alloys: Electrodeposition of Rare Earth Element-Based Thin Films—State of the Art

**DOI:** 10.3390/ma19071350

**Published:** 2026-03-28

**Authors:** Ewa Rudnik

**Affiliations:** Faculty of Non-Ferrous Metals, AGH University of Krakow, Mickiewicz Ave. 30, 30-059 Krakow, Poland; erudnik@agh.edu.pl

**Keywords:** electrodeposition, rare earth metals, lanthanides, ionic liquid, deep eutectic solvent, molecular organic, magnetic properties, corrosion, hydrogen evolution catalyst

## Abstract

The electrodeposition of rare earth metal alloys has attracted considerable interest, not only due to the challenges associated with the reduction in metal ions, but also because of their unique material properties and promising technological applications. This review presents a comprehensive analysis of the state-of-the-art in the electrochemical deposition of these alloys, focusing on various electrolytic systems, including aqueous solutions, organic molecular solvents, ionic liquids, and deep eutectic solvents. Despite inherent problematic factors such as low reduction potentials, competing hydrogen evolution reactions, and difficulties in controlling metal formation, recent advancements have enabled improved control over film formation, typically through the induced codeposition of lanthanides with iron-group metals. The influence of key factors, such as electrolyte composition and current/potential modes, on alloy codeposition, elemental and phase composition, structure, and deposition efficiency is discussed. The magnetic properties, electrocatalytic behavior, and corrosion resistance of the deposited films are also shown, highlighting their relevance for high-performance applications.

## 1. Introduction

Electrodeposition is a highly versatile and scalable technique for the fabrication of thick coatings, thin films, and a wide range of micro- and nanostructured materials [1,2]. The specific properties of electrodeposited thin metallic films originate from their growth mode, which is strongly governed by the substrate, e.g., through epitaxial growth with reproduction of the substrate texture and crystallographic orientation, while grain development is controlled by the early stages of nucleation. Consequently, thin films, with thicknesses ranging from nanometers up to only a few micrometers, are particularly important for functional applications determined by surface-related properties. In contrast, the growth and bulk structure of thick layers are governed by steady-state conditions at the cathode surface, resulting in coatings with thicknesses ranging from several tens to several hundreds of micrometers. This makes them suitable for protective applications, such as corrosion and wear resistance.

The deposition process is governed by electrochemical reactions driven by an applied current, resulting either in (i) the direct reduction of metal ions to their atomic form or (ii) the formation of metal compounds through subsequent chemical steps caused by interfacial changes at the substrate surface. Such reactions can be carried out in aqueous, non-aqueous, or molten salt electrolytes. When combined with the operating under different current or potential regimes, electrodeposition offers extensive possibilities for tailoring multicomponent alloys and compounds with diverse elemental compositions, structures and properties.

**Figure 1:**
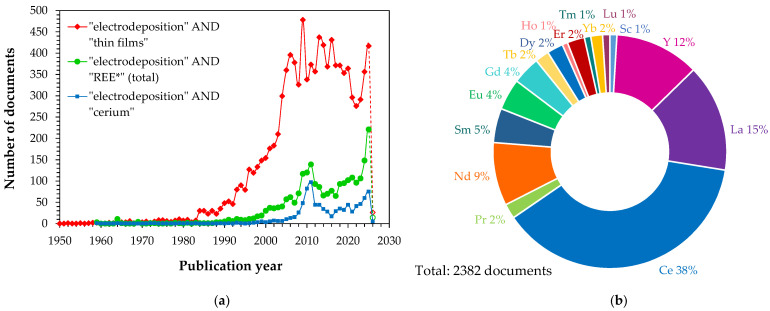
Scopus-indexed publications (1950–2026): (**a**) number of documents retrieved using the keyword combinations “electrodeposition” AND “thin films”, “electrodeposition” AND “name of rare earth element*” (total number of records) or “electrodeposition” AND “cerium”; (**b**) distribution of documents by individual metals based on the search query “electrodeposition” AND “name of rare earth element”. Data source: Scopus, Elsevier [3] (19 January 2026).

Interest in the application of electrodeposition techniques for the formation of thin films is reflected in the growing number of scientific publications since the late 1980s. This trend is illustrated by publication statistics compiled from the Elsevier Scopus database (Figure 1a). At the same time, growing scientific attention has been directed toward the electrodeposition of rare earth element thin films. A bibliometric analysis of the Scopus database using the keyword combination “electrodeposition” AND “the name of an individual rare earth metal” indicates that a record number of 221 documents addressing this topic were published in 2025 alone (Figure 1a). Notably, publications related to cerium account for the largest share (Figure 1a,b), representing about 38% of the total (1960–2026). This predominance can be attributed to the widespread use of cerium as a component of conversion coatings, as a reinforcing phase in electrodeposited composites, or as a dopant in magnetic and catalytic materials. Electrodeposits containing lanthanum, yttrium, and neodymium represent another important area of scientific interest (Figure 1b).

Bibliometric analysis highlights rare earth elements (REE; i.e., lanthanides, scandium, and yttrium) as critical and strategic components [4]. This reflects their importance in advanced materials due to the unique functional properties of their alloys and compounds, such as luminescence [5], catalytic activity [6,7], magnetic behavior [8], and enhanced corrosion and wear resistance [9]. These characteristics make REE-containing thin films particularly valuable for high-tech applications in electronics, renewable energy technologies, and protective coatings [10].

Thin metal films can be produced by a variety of chemical and physical methods [11]. Among them, the electrodeposition provides a flexible, low-temperature, and resource-efficient alternative to conventional vacuum techniques [12]. The electrochemical techniques allow rare earth elements to be incorporated into metallic or compound thin films with precise control over composition and microstructure, while ensuring good adhesion and uniform thickness. It should be emphasized, however, that the electrochemical behavior of REEs is challenging due to their highly negative reduction potentials [13], the formation of multiple chemical complexes in solution [14,15], and strong interactions with co-deposited transition metals, ionic species, or some substrates (in molten salts) [16,17]. These factors make the direct deposition of metallic REEs difficult and have stimulated the development of novel electrolytes, mainly non-aqueous systems, as well as induced-codeposition strategies for alloy formation [18]. In contrast, conventional aqueous electrolytes facilitate the straightforward formation of REE compounds, often with semiconducting properties [19].

Diversity of electrolytes and deposition strategies offers a wide range of possibilities for tailoring the composition, structure, and functionality of REE-based thin films. Therefore, this review provides a comprehensive overview of approaches for the electrodeposition of rare earth element alloys, with particular focus on their deposition modes and mechanisms as well as resultant material characteristics.

## 2. Electrolytes

Rare earth metals occur in solution predominantly as stable trivalent cations, although some elements may also form divalent or tetravalent species. These ions can undergo redox transformations to metallic or lower oxidation states, characterized by standard reduction potentials summarized in Table 1. The standard potentials of the M/M^3+^ couples are highly negative, typically in the range of about −2.0 V to −2.4 V versus the standard hydrogen electrode SHE [20]. As a result, during electrolysis of aqueous electrolytes, hydrogen evolution occurs preferentially, effectively suppressing the direct electrodeposition of metallic rare earth elements under conventional conditions. Consequently, a sequence of interrelated processes leads to the formation of secondary hydroxide/oxide/hydroxy salt products [13,21,22,23] at the cathode surface:

2H_2_O + 2e → H_2_ + 2OH^−^ (1)

M^3+^ + 3OH^−^ → M(OH)_3_ (2)

2M(OH)_3_ → M_2_O_3_ + 3H_2_O (3)

Although early studies reported the electrodeposition of rare earth metals from aqueous solutions, such as lanthanum [24] or samarium [25,26], the formation of a metallic phase was not definitely confirmed, as white-gray deposits were characterized mainly by visual and microscopic observations, and no results of phase composition analysis were provided. Nevertheless, the deposition potential was found to depend on the substrate type (copper, brass, titanium, stainless steel, ITO-coated glass) as well as on the complexing agents added to the bath (oxalate, citrate, thiocyanate, EDTA, tartrate). Other reports did not confirm the formation of single-metal electrodeposits from aqueous solutions, as observed in the case of terbium [27], samarium [28], and other rare earth elements [13,21].

The electroreduction behavior of rare earth metal ions in aqueous electrolytes can be significantly modified during alloy codeposition, particularly with iron-group metals such as iron, nickel, and cobalt [16,29], as they show high cathodic overpotential. This phenomenon has been observed in both simple salt solutions and complex salt electrolytes. Some mechanisms have been proposed to explain this phenomenon and these are discussed in the further sections of this article.

**Figure 2:**
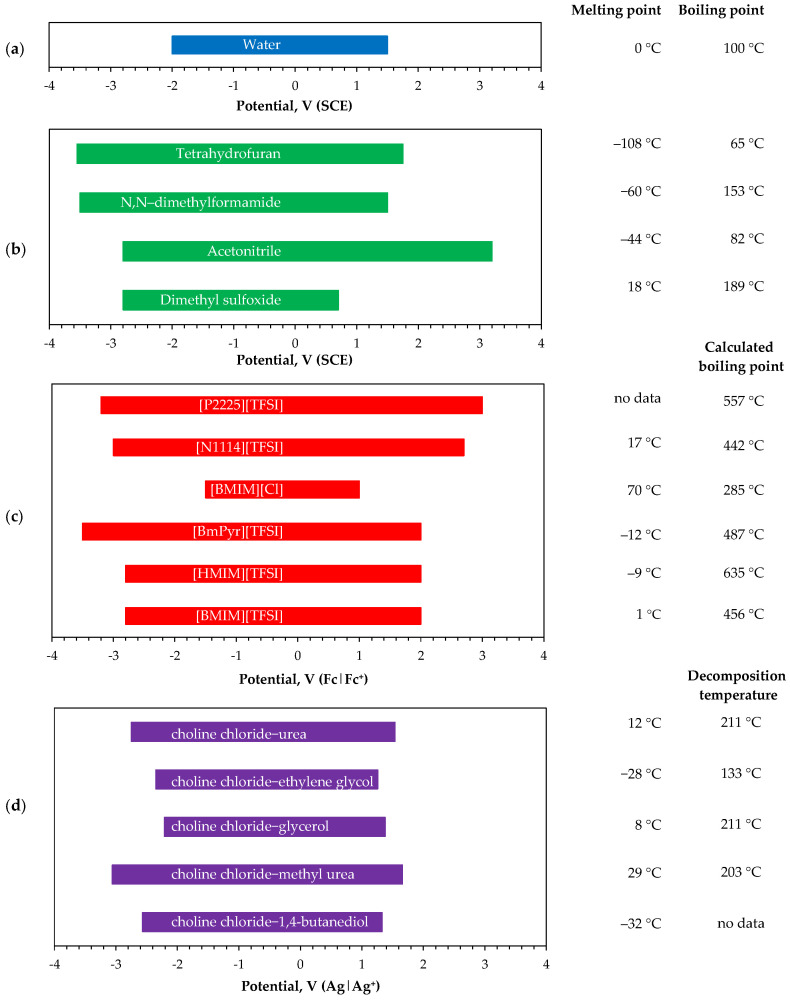
Electrochemical windows, melting and boiling points of solvents: (**a**) water, (**b**) molecular organics, (**c**) ionic liquids (abbreviation explanation at the end of the article), and (**d**) deep eutectic solvents. Data taken from [15,20,34,35,36,37,38,39].

Organic electrolytes offer an effective route to overcome the limitations of aqueous systems, facilitating the electrodeposition of rare earth metals [18,30,31,32,33] and their alloys [16,17,18]. This category includes molecular organic solvents, ionic liquids, and deep eutectic solvents. These media provide significantly wider electrochemical windows than water (Figure 2), thereby enabling the reduction of metal ions at highly negative potentials.

Molecular solvents are conventional non-aqueous media composed of polar organic compounds, both aprotic (e.g., tetrahydrofuran THF, N,N-dimethylformamide DMF, formamide FA, dimethyl sulfoxide DMSO, acetonitrile AN, acetone AC) and protic (e.g., ethanol, methanol) [34]. These solvents are relatively inexpensive, readily available on an industrial scale, and exhibit advantageous physicochemical properties, such as low viscosity, high metal salt solubility, and acceptable thermal and electrochemical stability. Notably, the half-wave potentials, which are directly related to the reduction potentials for lanthanide M/M^3+^ couples, are strongly dependent on the solvent, increasing in the order DMSO < DMF < AC < AN [40]. Molecular organic liquids are used for the electrodeposition of both metallic phases and chemical compounds.

Ionic liquids IL consist of large organic cations (e.g., pyridinium, pyrrolidinium, quaternary phosphonium, piperidinium, imidazolium, and quaternary ammonium) combined with smaller organic (e.g., triflate TFO and triflimidate TFSI) or inorganic (e.g., chloride, bromide, sulfate, and hexafluorophosphate) anions [41]. These compounds exhibit a wide liquid range, with melting points below 100 °C and no well-defined boiling points due to thermal decomposition [37], but they possess chemical and thermal stability sufficient for electrodeposition processes. Ionic liquids show high solubility for metal salts, as well as moderate-to-high electrical conductivity (up to 0.1 S/m) and broad electrochemical windows (2–8 V) [15,36]. The main drawback of these solvents is their complex synthesis and purification methods [41], which make ionic liquids relatively expensive. Additionally, they are sensitive to water, requiring a controlled atmosphere during use for electroplating purposes. Ionic liquids are used principally for the electrodeposition of metals and alloys rather than chemical compounds.

Deep eutectic solvents DES are eutectic mixtures of two or more molecular compounds, in which one component acts as a hydrogen-bond acceptor (e.g., choline halides) and the other as a hydrogen-bond donor (e.g., urea or alcohols). They exhibit melting points lower than those of their individual components and lower than that of an ideal liquid mixture [38]. DESs share many advantageous physicochemical properties with ionic liquids, including a suitable liquid range, low volatility, and high solubility for both inorganic and organic compounds. Their simple preparation by mixing the components with moderate heating makes them significantly easier and more cost-effective to produce. However, DESs are more viscous than conventional aqueous or molecular solvents and are not stable at elevated temperatures, with progressive volatilization and decomposition starting at 200–250 °C [39]. These systems are typically used for metal phase electrodeposition, but they exhibit relatively low deposition rates, often an order of magnitude lower than conventional aqueous baths. To enhance electroreduction, the operating temperature is usually maintained slightly higher to reduce viscosity and improve bath conductivity. DESs also have limited throwing power, making it difficult to achieve uniform deposition thickness, and their properties deteriorate at elevated water contents, which is particularly important when depositing reactive metals [42].

It is also worth noting that electrolysis with insoluble anodes (e.g., platinum, glassy carbon, and tungsten) can be problematic due to the electrochemical decomposition of components in organic solvents, including both ILs and DESs [42,43,44]. This can change the speciation of the bath and affect the stable width of the electrochemical window. Moreover, anodic oxidation of metal ions with multiple oxidation states may have unfavorable effects, as observed in some aqueous solutions [1]. These effects cannot be ignored over longer operation times, as they may modify cathodic reactions and affect the quality of the cathode deposits. Such phenomena, however, need detailed studies.

Finally, it is worth mentioning molten salts used as electrolytes, typically eutectic mixtures of alkali metal (e.g., sodium, cesium, and lithium) or alkaline earth metal (e.g., calcium and magnesium) chlorides or fluorides [45,46,47]. Although they are generally not applied to thin-film production, molten salts represent a common method in the final stage of rare earth element production [47] and constitute a potential way for the recovery of rare earth elements from spent nuclear fuel [46]. These systems operate at high temperatures, typically 500–1100 °C. Unlike aqueous solutions, molten salt electrolytes allow rare earth metals to form both stable and soluble divalent and trivalent ionic species. The electrodeposition of rare earth metals from such electrolytic baths strongly depends on the nature of the cathode substrate. In single rare earth metal systems, deposition does not occur on inert cathodes (e.g., molybdenum and tungsten) but proceeds only on reactive cathodes (e.g., nickel, cobalt, copper, zinc, and magnesium) with simultaneous alloy (intermetallic phases) formation. In contrast, the intentional introduction of a rare earth salt together with aluminum, magnesium or transition metal salt promotes alloy formation even on inert substrates. Alloy formation during reactive electrodeposition is driven by the thermodynamic tendency to generate intermetallic compounds present in binary equilibrium phase diagrams [45,47]. This results in a shift in the codeposition potential toward more electropositive values compared to pure metal deposition. In turn, inert substrates do not spontaneously form intermetallic compounds with rare earth metals; thus, the high cathodic overpotential of the latter prevents their deposition. Instead, only a partial reduction in trivalent ions to divalent species is observed.

## 3. Electrodeposition of Alloy Films

### 3.1. Aqueous Baths

Aqueous solutions used for the electrochemical formation of rare earth alloys are typically acidic and often contain complexing agents. Metal ions are introduced as chloride, sulfate, or sulfamate salts, while nitrates are occasionally used. Various electrodeposition modes can be employed, including potentiostatic, galvanostatic, or pulsed current techniques at ambient temperature. Electrodeposition conditions for selected thin-film alloys of rare earth elements are summarized in Table 2.

**Figure 3:**
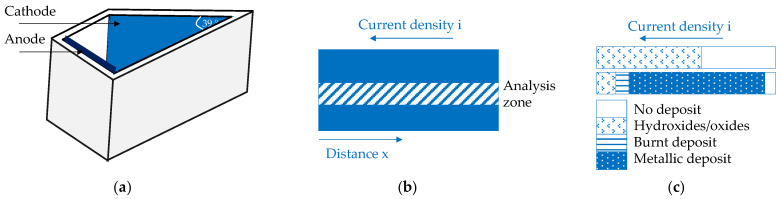
Scheme of the following: (**a**) Hull cell, (**b**) cathode surface with analysis zone, and (**c**) data interpretation. Based on [57].

Hull cell measurements were used as a fast and simple method for determining the conditions required for metallic phase deposition [57]. The trapezoidal geometry with angled electrodes (Figure 3a) generates a continuous current density gradient along the cathode (Figure 3b). This allows us to correlate deposit appearance, thickness, and composition with the local current density and the bath formulation used (Figure 3c). The primary current density distribution can be estimated using the following relationship:

i = I∙(5.1 − 5.24∙log x)(4)

where i—current density; A/dm^2^; I—applied current intensity; A; x—distance on the cathode surface measured from the edge nearest anode (i.e., from left to right; Figure 3a,b).

Gong and Podlaha [27] analyzed the formation of Fe–Tb alloys as a function of the dimensionless position along the cathode surface at different deposition times (0.5–4 h) using the Hull cell. They observed that the terbium content remained relatively constant along the cathode surface, typically between 10 and 20 wt%, regardless of the deposition time. In turn, the deposit thickness increased with plating time in two distinct regimes, namely below (up to 0.1 µm), and above 2 h (0.2–0.7 µm). Bright gray layers were formed over approximately half of the cathode area near the low current density region. The estimated current efficiencies were very low, in the range of 0.5–2%. It was also observed that the Fe^2+^ reduction potential shifted toward more positive values during codeposition compared to iron deposition alone. It was concluded that terbium codeposition proceeds via an induced mechanism, and the formation of a mixed-metal reaction intermediate was suggested, although no detailed mechanistic explanation was provided.

Further studies [48] on Co–Tb codeposition from simple salt solutions showed that alloy formation occurs only within a narrow solution pH window of 1.5–2.2 (potentiostatic deposition at −3 V). Under more acidic conditions, reduction in Tb^3+^ ions was completely inhibited, whereas at pH 2.5–3, the electrolyte became unstable and precipitation occurred. Under optimal conditions, high-terbium alloys were obtained, with terbium content exceeding 50 wt%. Importantly, the codeposition mechanism was proposed as a coupled induced-anomalous process, in which terbium reduction is induced by cobalt ions (induced codeposition):

Tb^3+^ + H_2_O → TbOH^2+^ + H^+^ (5)

TbOH^2+^ + Co^2+^ + e → TbCo_ads_ + OH^−^ (6)

TbCo_ads_ + 2e → Tb + Co^2+^ (7)

while cobalt deposition is inhibited in the presence of terbium ions, similarly to the behavior observed in anomalous codeposition.

Fukami et al. [58] studied the electrochemistry of Co–Tb codeposition using flat and nanoporous electrodes. They found that terbium incorporation promotes hydrogen ion reduction, leading to terbium oxide formation and its incorporation within the amorphous cobalt layer. The authors concluded that, although studies on metal alloys composed of rare earth and iron-group metals have been reported, no metallic alloy formation was detected in their work.

Schwartz et al. [28] electrodeposited a series of binary alloys composed of iron-group metals and rare earth elements (Nd, Gd, and Sm) from sulfamate baths containing amino acids as complexing agents. Galvanostatic deposition (0.2–4 A/dm^2^) produced cracked, nanocrystalline, or amorphous deposits, with varying tendencies for rare earth metal incorporation. Nd and Gd contents in the deposits followed the order Ni > Fe > Co, whereas for Sm the trend was Ni > Fe = Co. In addition, iron-containing metallic deposits could be obtained only up to 1.5 A/dm^2^, while cobalt- or nickel-based deposits remained metallic at current densities above 3 A/dm^2^. The use of organic additives enhanced lanthanide incorporation in the order glycine > serine > alanine. Detailed analysis of codeposition in the presence of glycine led to the proposed mechanism in which the dipolar nature of the amino acid promotes the formation of heteronuclear coordination complexes (e.g., M^2+^REE^3+^(Gly)_2_(HGly^±^)^3+^), enabling stepwise reduction in surface-oriented divalent ions (M^2+^, i.e., Ni^2+^, Fe^2+^, Co^2+^) and trivalent ions (REE^3+^, i.e., Nd^3+^, Gd^3+^, Sm^3+^) via atomic hydrogen and/or direct electron transfer at the cathode, resulting in alloy formation. The current efficiencies of alloy deposition decreased with increasing current density, and the highest values obtained were in the range of 25–30%.

More detailed studies on Co–Sm alloys produced from sulfate–glycine baths [29,49] showed that the organic additive extends the current density range for deposition of amorphous metallic phase by preventing the formation of hydroxide or burnt deposits (Hull cell experiments). The latter was further suppressed by the addition of a supporting electrolyte (ammonium sulfamate or chloride) or by increasing the temperature (to 60 °C). In contrast to direct current deposition, pulsed current deposition did not result in increased samarium content with increasing temperature, while samarium incorporation was enhanced by shorter on-times at a constant duty cycle [29]. Long et al. [50] used a similar bath formulation, but Co–Sm alloy deposition was carried out under potentiostatic conditions. High-samarium coatings were obtained, with their maximum contents (20–43 at%) observed at an electrodeposition potential of −1.9 V (SCE) for different Co^2+^/Sm^3+^ concentration ratios in the bath and temperatures of 5 or 35 °C. The amorphous films were subsequently annealed, resulting in the transformation to the Sm_5_Co_17_ phase. This significantly improved the coercivity of the alloys (from 7 to 10 kA/m to 33–38 kA/m).

Lou et al. [51] examined the influence of an external magnetic field on the electrodeposition of Fe–Sm alloys. The presence of the magnetic field affected the evolution of the metallic phase, leading to the formation of finer-grained coatings, with a more pronounced refinement observed under the perpendicular field configuration compared with the parallel one. At the same time, increasing magnetic field strength in the parallel configuration hindered samarium incorporation, reducing its content in the deposits from 4.5 at% at 0 T to 3 at% at 4 T, while no comparable effect was detected in the perpendicular configuration. In the absence of a magnetic field, the Fe–Sm coatings consisted primarily of Fe and Sm_2_Fe_17_ phases. The application of a magnetic field substantially enhanced the contribution of the SmFe phase, and under certain conditions, the formation of the metal oxide phase Sm_3_Fe_5_O_12_ was observed.

Wang et al. [52] synthesized ternary alloys Ni–Ce–P and Ni–Nd–P using a chloride–citrate bath with H_3_PO_3_ and NaH_2_PO_2_ as phosphorus sources. Although similar amounts of rare earth elements were incorporated (about 34 wt%), the phosphorus content was higher in the neodymium-containing alloy (11.5 wt% P) than in the cerium-containing alloy (7.6 wt% P). The deposits were amorphous, but upon annealing, they exhibited a glass transition temperature of 385 °C.

**Figure 4:**
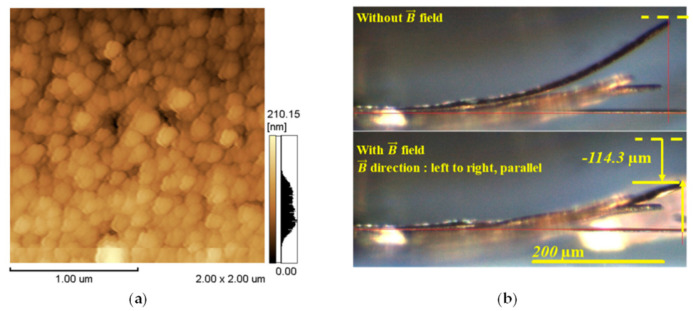
AFM image of an electrodeposited TbDyFe thin film (**a**) and optical image showing magnetostrictive actuation of a Si–TbDyFe bi-material cantilever (**b**). Reprinted from [53] under License CC BY.

Shim et al. [53] electrodeposited Tb_x_Dy_1−x_Fe_y_ thin films (200–300 nm) from a chloride–sulfate–tartrate–citrate bath at a constant potential. They observed an increase in the iron content (40–80 at%) over a relatively narrow potential range (−0.92 V to −0.95 V), accompanied by a decrease in terbium and dysprosium contents, although their relative shares remained comparable. The deposits were fine-grained polycrystalline films (Figure 4a), but the grain size was larger than that of sputtered films. The films exhibited magnetostrictive properties of 1250 ppm (Figure 4b) and an energy density of 100–165 kJ/m^3^, nearly equivalent to bulk Terfenol-D. This demonstrated the high potential of the obtained material for applications in magnetic actuators, energy harvesting, and sensors.

Faltas et al. [54] investigated the electrodeposition process and magnetic properties of films produced in a Ni–Co–Fe–sulfate bath with the addition of terbium and/or dysprosium salts at various concentrations. The quaternary and quinary films contained 86–92% Ni, 2–3% Co, 5–9% Fe, and less than 1% Tb or Dy. Despite these small amounts, the lanthanides affected the lattice parameter and crystalline strain of the alloys. Although the resulting films did not exhibit magnetostriction as high as Terfenol-D (1400 ppm [53]), with values of only about 370 ppm, they achieved saturation magnetostriction at much lower magnetic fields (20 kA/m vs. 160 kA/m), resulting in easier magnetization and demagnetization behavior.

Liu [55] synthesized novel nickel-based alloys containing cerium, praseodymium, and/or holmium as potential catalysts for hydrogen evolution. A series of ternary and quaternary alloys deposited in one step on nickel foams was characterized in terms of their microstructure and electrochemical properties. In addition to lanthanides, the deposits contained 1–4 wt% oxygen, which was attributed to the presence of CeO_2_ (XPS analysis). In turn, X-ray diffraction revealed the formation of a CeNi_3_ phase within an otherwise predominantly amorphous matrix. The Ni–Ce–Pr–Ho coatings exhibited high electrocatalytic activity in alkaline media (1 M KOH), corresponding to a low overpotential of 78 mV at 1 A/dm^2^, along with high durability for long-term performance (24 h).

Gandhi et al. [56] described the galvanostatic electrodeposition of ternary Ni–Fe–Sm alloys from sulfate electrolytes and observed that, despite the absence of distinct samarium-rich phases, increasing samarium incorporation led to a reduction in both nickel and iron contents. This compositional shift was associated with pronounced microstructural changes, including grain coarsening from 71 nm in Sm-free deposits to 156 nm at 25 at% Sm, a decrease in lattice strain from 0.23% to 0.14% over the same composition range

Finally, it is worth noting that lanthanide ions introduced into the plating bath in small amounts, although they are not electrochemically reduced, still influence the morphology and structure of the main metal. For example, lanthanum or cerium salts have been used as additives in nickel plating baths [59,60]. Lanthanum is typically not incorporated into nickel coatings in readily detectable amounts (although it can form the Ni_7_La_2_ intermetallic phase), but it improves deposit compactness by refining grain structure and modifying the nickel preferential plane orientation, which results in increased corrosion resistance and hardness [59]. Cerium exhibits a similar behavior, but the effect is weaker [60]. The structural modification of nickel is attributed to the adsorption of lanthanide cations at the cathode surface, which act as surface-active species that inhibit nickel ion reduction and thereby affect metal nucleation and growth. López et al. [61] showed by XPS analysis that the codeposition of nickel with samarium (1.9%) resulted in the incorporation of the latter in oxide forms of both trivalent and divalent states. This shows two-step reduction of samarium(III) involving one- followed by two-electron transfer reactions. This also suggests that the generation of OH^−^ ions at the cathode surface (Equation (1)) leads to the formation of samarium hydroxides (Equation (2)), which are subsequently incorporated into the nickel matrix.

**Figure 5:**
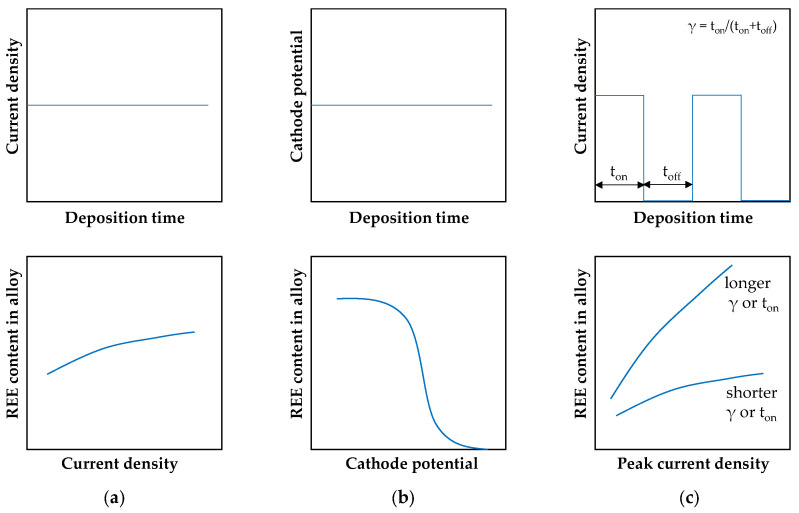
Electrodeposition modes of binary alloys and general trends in rare earth element incorporation: (**a**) galvanostatic deposition, (**b**) potentiostatic deposition, and (**c**) pulsed current deposition. Based on [28,29,48,49,50,51].

The mentioned examples indicate that the electrodeposition of alloys containing mainly lanthanides is possible; however, only iron-group metals are capable of inducing the reduction of more electrochemically active rare earth ions. Undoubtedly, the formation of hydroxide-type intermediate species plays an important role in this process, although such intermediates have not been unambiguously identified. Depending on the electrolysis conditions (Figure 5), from trace amounts up to several tens of percent of the alloying element can be incorporated. Nevertheless, many studies on alloy deposition from aqueous systems have not demonstrated clear codeposition of rare earth elements in the metallic state, instead identifying predominantly amorphous materials. The grain-refining effect induced by the presence of such alloying elements was evidenced. In addition, current efficiencies are only rarely reported, which makes it difficult to reliably assess the effectiveness of rare earth incorporation and the true efficiency of the alloy deposition process. The electrodeposition process is most commonly carried out at ambient temperature, with moderately elevated temperatures (40–60 °C [49,54]) applied in some cases.

### 3.2. Molecular Organic Baths

The electrodeposition of rare earth elements containing alloys from molecular organic solvents has been an established practice [62,63,64,65], largely due to the limited availability in the past (1990s–2000s) of more advanced conductive organic solvents such as ionic liquids or deep eutectic solvents. Among molecular solvents, DMSO was the most widely used in electrolysis because its wide electrochemical stability window allows the codeposition of metals with highly different standard potentials, thus enabling the formation of REE-containing alloys at ambient temperature with iron-group metals (as in aqueous systems) and also with other elements (Table 3). Cobalt was most frequently selected as the base alloy metal due to its intrinsic magnetic properties, which are further enhanced by lanthanide incorporation.

Molecular solvents readily dissolve metal salts; however, the electrodeposition process requires that the salts be entirely anhydrous or that the electrolyte solution be dehydrated using molecular sieves. Typically, organic electrolytes consist of simple inorganic metal salts, although in some cases, various additives are introduced to achieve specific functions. These include supporting electrolytes to increase the conductivity and lower the freezing point of DMSO-based solutions (e.g., (n-Bu)_4_NBF_4_ [68,69], LiClO_4_ [70,71,77], LiCl [64,66,74,75,76,78], urea [67,73]), and compounds that promote film formation instead of powdery deposits (e.g., ethylenediamine [62]).

An et al. [72] compared the suitability of organic solvents (DMF, DMSO, acetonitrile, ethanol, and formamide) for the codeposition of lanthanum with nickel. Among the solvents studied, DMF exhibited the most advantageous characteristics, including high La(NO_3_)_3_ solubility (in contrast to the low solubility observed in DMSO), good bath stability (ethanol-based baths were unstable due to solvent volatility), favorable coating morphology (porous layers were formed in acetonitrile), and a high lanthanum content in the deposited alloy (in formamide-based electrolytes, Ni–La alloy formation was possible only at pH < 1). The effect of complexing agents was also examined in DMF baths, revealing that despite their limited solubility, EDTA and nitrilotriacetic acid improved coating quality and enhanced lanthanum incorporation, while ammonium bifluoride increased the lanthanum content by a factor of 4–6. Notably, fluoride ions also increased the solubility of NiCl_2_. In turn, ammonium citrate improved coating quality but suppressed lanthanum incorporation. The influence of substrate material (copper, brass, stainless steel, titanium, and titanium alloy) on alloy formation from DMF-based baths was evaluated, with reported current efficiencies of 53–65% and lanthanum contents of 10–21 wt%. The Ni–La coatings were bright and amorphous, although XPS analysis indicated that lanthanum was present predominantly as LaH_2_, with smaller fractions occurring as oxides and metallic lanthanum within the nickel matrix.

**Figure 6:**
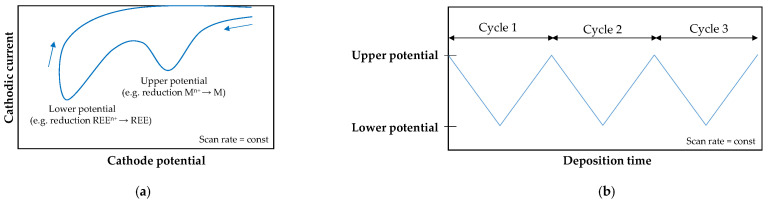
Cyclic electrodeposition: (**a**) cyclic voltammetry (arrows indicate the scan direction) and (**b**) applied potential mode.

Similarly to aqueous systems, electrodeposition was performed under potentiostatic [64,65,66,67,68,69,76,79], galvanostatic [62,72], or pulsed current conditions [63,77]. In molecular organic media, however, an additional method was introduced [67,70,73,74,75,80], based on cyclic changes in the potential between defined upper and lower limits at a controlled sweep rate (Figure 6). Li et al. [67] compared the codeposition of cobalt with cerium from a urea–DMSO bath under constant and cyclic potential regimes. Both modes produced smooth, uniform, amorphous deposits with a metallic luster and comparable cerium contents. Under cyclic conditions, the sweep rate markedly affected coating adhesion, which was poor at 10 and 100 mV/s but improved at intermediate values. Yuan et al. [70,71] studied the deposition of Co–Tm alloys and showed that, during potentiodynamic (cyclic) deposition, thulium incorporation depended strongly on cycle duration and the number of cycles. When the cycle time was fixed at 20 min, increasing the sweep rate from 2 to 20 mV/s had little influence on Tm content (4–5 wt%), whereas at a constant number of cycles (five), the same increase in sweep rate resulted in an evident decrease in Tm content (from 13 to about 3 wt%).

**Figure 7:**
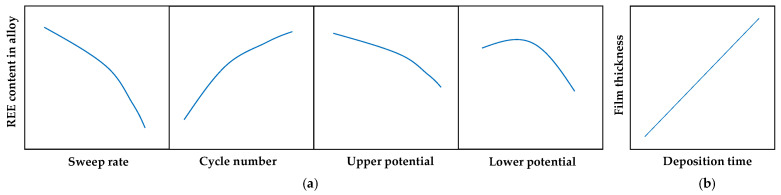
Cyclic electrodeposition of ternary alloys: (**a**) general trends in rare earth element incorporation and (**b**) time-dependent film thickness. Based on [74,75,78].

For ternary alloys, direct comparison of current- and potential-controlled modes remains difficult due to the lack of systematic studies conducted under identical deposition conditions, such as bath composition and temperature. Nevertheless, similar trends can be identified across different ternary systems deposited under cyclic potentiodynamic conditions: (i) increasing sweep rate reduces REE content [74,75,78], (ii) increasing the number of cycles tends to enhance REE incorporation [74,75,78], (iii) shifting the upper potential to more negative values leads to lower REE content [74,75] (but may also increase [73]), (iv) more negative lower potentials may result in either a maximum [74,75] or a minimum [73] in REE content, and (v) film thickness is a linear function of deposition time [74,78] (Figure 7).

Regardless of the deposition technique and operating conditions, the as-deposited coatings are amorphous. Distinct intermetallic phases are detected only after post-deposition heat treatment (500–780 °C), predominantly M_2_REE-type intermetallics (e.g., Co_2_Ce [67], Co_2_Dy [74], Co_2_Lu [76], Bi_2_Yb [66]). Other phases such as Co_3_Gd [69], Co_5_Ce, and Co_19_Ce_5_ [67], LuNi [64,68], and LuBiNi [68] were also identified.

In DMF or DMSO media, metal ions are generally assumed to exist as large solvent-coordinated complexes, most commonly of the type [M(DMF)_n_]^3+^ [69,79] or [M(DMSO)_n_] [64,67,74], with coordination numbers in the range 1–6 [64,74] or even up to 8–12 [67,69]. Certain lanthanide ions are reduced in a single electrochemical step directly from the trivalent state to the metallic form (e.g., ytterbium [66], cerium [67], gadolinium [69], thulium [70], and lanthanum [77]), whereas others undergo a two-step reduction pathway (e.g., europium [68], samarium [79]). Despite these observations, the mechanism underlying alloy codeposition remains only partly understood. In some cases, the process has been identified as proceeding via an induced codeposition mechanism (e.g., Ni-Lu [64] and Ni-La [72]), in which lanthanide ions appear to inhibit the electrodeposition of iron-group metal ions, while the latter, in turn, promote the deposition of lanthanide.

The examples shown indicate that the electrodeposition of REE-containing alloys from molecular organic-type baths is relatively straightforward. The iron-group metals are generally required as the base component enabling alloy formation, while the underlying mechanism of codeposition is rarely discussed. The electrodeposition process is most commonly carried out at 25–35 °C. Depending on the electrolysis conditions, from several to several tens of percent of the alloying REE can be incorporated, with the formation of the amorphous deposits. Current efficiencies were not reported, which makes it difficult to reliably assess the process and to compare these systems with other electrolyte formulations.

### 3.3. Ionic Liquid Baths

The electrodeposition of rare earth elements from ionic liquids has been extensively investigated [15,18]. However, their codeposition as alloys has only begun to attract research attention over approximately the past two decades [16], and such attempts have not always been successful (Table 4).

In contrast to the two previously discussed electrolyte systems, it is difficult to generalize the influence of current- or potential-controlled deposition modes on rare earth incorporation into alloy films, as most studies have focused on codeposition under cyclic voltammetric conditions [80,81,82,83,85,87,88,89], while only a few provide systematic investigations conducted under well-defined parameters [84,86]. Yang et al. [86] examined the codeposition of nickel and lanthanum from a chloride–[EMIM][Cl]–ethylene glycol electrolyte and demonstrated that La^3+^ reduction is induced by nickel, whereas lanthanum does not deposit alone. Alloy formation followed an instantaneous nucleation model, similar to that observed for pure nickel. Lanthanum incorporation was enhanced by increasing both current density (up to 1 A/dm^2^) and temperature (80–100 °C). As the lanthanum content increased, the deposits evolved from a single-phase fcc solid solution (4 at% La) to an amorphous structure at higher contents (9.5 at% La), accompanied by a contraction of the nickel lattice parameter that deviated from Vegard’s law. In turn, Xu et al. [84] applied pulsed current deposition to produce Fe–Nd alloys from a chloride–[EMIM][DCA] system and found that the pulse “on” time (10–100 ms) had little effect on neodymium incorporation, whereas increasing current density enhanced its content. Additionally, lower Fe^2+^ concentrations in the electrolyte promoted higher neodymium incorporation under both conditions. An induced codeposition mechanism was identified in this system and attributed to the formation of a transition-state iron intermediate facilitating the simultaneous deposition of both metals. A similar effect was observed in the Pr–Bi system, where praseodymium alone did not reduce at the cathode, while the addition of bismuth salts promoted deposition through the initial formation of a thin metallic binding layer [80].

The cathodic current efficiencies for alloy deposition are relatively low, typically in the range of 60–70% [84,87]. An et al. [87] reported that the current efficiency of Dy–Tb codeposition increased with temperature, current density, and metal salt concentration, and beyond a certain threshold, no further improvement was observed with the efficiency stabilization at a maximum value of about 75%.

**Figure 8:**
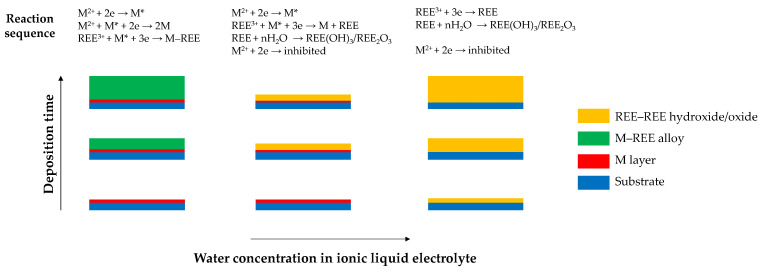
Effect of water in ionic liquid on codeposition of iron-group (M) and rare earth (REE) metals (based on [83]).

Molodkina et al. [82,83] investigated the effect of water addition to the ionic liquid [BmPyr][DCA] on alloy formation and inhibition phenomena in Co–Sm and Fe–Nd systems. On a Pt substrate [83], three distinct deposition regimes were identified depending on water content: (i) effective Nd–Fe codeposition at low water concentrations (12–74 mM), (ii) partial blocking of the cathode surface at intermediate water levels (74–800 mM), and (iii) complete surface passivation at higher water contents (above 500 mM) due to precipitation of neodymium hydroxides and oxides (Figure 8). In both systems [82,83], an induced codeposition mechanism was proposed, in which a reduction in the iron-group metal generates activated species M* that facilitate the reduction in the rare earth element to its metallic form by shifting its reduction potential toward more positive values. The presence of water, however, promotes oxidation of metal species, leading to the concurrent formation of oxide and hydroxide phases during electrodeposition. The resulting oxide/hydroxide films hinder transition-metal reduction and suppress formation of the active intermediate, causing an inhibition of the alloy codeposition.

Reported studies demonstrate that electrodeposited lanthanide-containing alloy films can exhibit structures ranging from crystalline to amorphous with increased alloying element (REE) concentration. However, systematic investigations addressing trends in the incorporation of alloy components under different current- and potential-controlled deposition modes remain scarce, which hinders the formulation of general conclusions. The presence of oxygen (up to several dozen percent) is consistently detected in the coatings [80,81,82,83,84,85,86,88], in addition to metallic [86,87] and intermetallic phases [88]. Moreover, although cathodic current efficiencies are only occasionally reported, they are typically below 80%, while the nature of the associated side reactions is not discussed and is most likely related to electrochemical decomposition of the solvent. The studies indicate that alloy formation is induced by iron-group metals. The presence of water suppresses the formation of metallic layers in favor of the precipitation of rare earth element hydroxides and oxides, highlighting the necessity of conducting the electrodeposition process under a strictly controlled atmosphere with very low humidity. Typical operating temperatures for electrodeposition from ionic liquids range from ambient conditions [83] to 80–110 °C [86], where elevated temperatures reduce electrolyte viscosity and promote enhanced ion mobility.

### 3.4. Deep Eutectic Solvent Baths

Deep eutectic solvents have recently attracted considerable interest for the electrodeposition of rare earth metal alloys. These electrolytes are typically low-temperature melts (operating up to about 70–80 °C) composed of anhydrous metal chlorides combined with urea and acetamide, as well as choline chloride-based mixtures with urea or ethylene glycol (Table 5).

In such systems, the deposition of pure rare earth metals cannot be achieved, and the process is induced by iron-group metals [90,91,92,93,95,98,100,101], leading to the formation of predominantly amorphous alloy layers. However, upon heat treatment, the deposits transform into crystalline materials in which various intermetallic phases can be formed (e.g., Co and Co_5_Gd [91], Co and CoLa [92], Co_17_Sm_2_ [93]).

Li et al. [90] electrodeposited three alloys with iron-group metals from urea–acetamide melts. In all cases, applying more electronegative deposition potentials increased the lanthanide content in the deposits with the scale of this effect followed the order Ni–La < Co–Gd < Fe–Sm for identical component concentrations in the electrolytes. Alloys with lower lanthanide contents were smooth, compact, and homogeneous, exhibiting no cracks, whereas higher alloying element contents resulted in increased surface roughness and reduced adhesion.

Panzeri et al. [95] examined the influence of glycine as a complexing agent on Co–Sm codeposition from choline chloride–ethylene glycol electrolytes with two different compositions (1:2 and 1:4.5). While a lower ethylene glycol content in the electrolyte enhanced samarium concentrations in the deposits, the presence of glycine further promoted samarium incorporation. The presence of glycine also improved deposit compactness and surface smoothness, although it resulted in fewer but wider cracks compared with glycine-free baths.

Murali Krishna et al. [98] investigated the electrodeposition of Ni–Sm alloys from a choline chloride–ethylene glycol–glycine electrolyte and observed a transition in the nucleation mechanism from progressive to instantaneous, occurring independently of the applied potential in the range −0.95 to −1.02 V on a glassy carbon substrate; however, no explanation for this phenomenon was provided. For comparison, Rabea et al. [96] demonstrated that in a calcium chloride hydrate–ethylene glycol system, Fe–Nd alloy nucleation proceeded instantaneously at lower overpotentials and changed into a progressive mode at higher overpotentials. This effect was attributed to differences in the nucleation kinetics of the alloy components.

**Figure 9:**
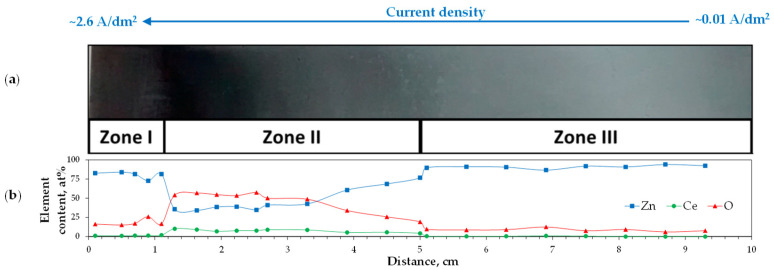
Visual appearance (**a**) and element distribution (**b**) along the cathodic deposit produced from a ChCl–U–ZnCl_2_–CeCl_3_·7H_2_O electrolyte in a Hull cell. Adapted from [99] under License CC BY.

Alloy deposits frequently contained oxygen [90,91,92,97,98,101], which may originate not only from the accidental air oxidation [91,92,101] or incorporation of electrolyte components (e.g., ethylene glycol), but can also be introduced through the formation of secondary hydroxide or oxide phases generated by water present in the electrolyte, such as in CaCl_2_·6H_2_O-based melt [96], through the use of hydrated metal salts [99], or as a result of trace contamination [98]. Marín-Sánchez et al. [99] prepared a choline chloride–urea bath containing anhydrous zinc chloride and cerium chloride heptahydrate, resulting in a final water content of 1.1 wt%. Deposits produced in a Hull cell exhibited distinct color zones (I—dark gray, II—dark blue, and III—light gray), depending on the local current density (Figure 9). These variations were correlated with different oxygen contents and with cerium incorporation in the form of mixed oxides, Ce_2_O_3_ and CeO_2_ (in a 1:1 ratio). Consequently, the study demonstrated that electrodeposition from deep eutectic solvents can yield nanocomposite materials rather than purely metallic alloys.

Li et al. [100,101] reported the electrodeposition of ternary alloy films containing praseodymium and magnesium together with cobalt or nickel from ChCl–U-based systems. They observed that the incorporation of both praseodymium (from 4 to 5 wt% to 11–12 wt%) and magnesium (from 1.5 to 3.5 wt% to about 7 wt%) increased with more negative deposition potentials, higher Pr^3+/^Mg^2+^ ratios, and longer electrolysis durations (up to 1 h) in the presence of cobalt [100]. The trends in nickel-based systems were also shown, where praseodymium incorporation reached a maximum depending on deposition time, potential, and the molar ratios of ions in the electrolyte [101].

Research studies indicate that electrodeposited lanthanide-containing alloy films from deep eutectic solvents exhibit amorphous structures and contain, in addition to metallic components, significant amounts of oxygen (up to several tens of atomic percent). Systematic investigations of trends in alloying element incorporation under different current- or potential-controlled deposition modes remain limited; however, higher current densities and more negative cathode potentials are expected to favor rare earth incorporation. Apart from the predominant potentiostatic and galvanostatic modes, alternative deposition techniques have not yet been explored. Alloy formation in DES is typically induced by iron-group metals, whereas the presence of water suppresses the formation of metallic layers in favor of rare earth hydroxides or oxides, highlighting the need for strictly controlled atmospheres with very low humidity. Cathodic current efficiencies were not reported. Electrodeposition from DES is usually conducted at moderately elevated temperatures (70–80 °C) to reduce viscosity and enhance bath conductivity.

## 4. Properties of Alloy Films

### 4.1. Magnetic Behavior

Electrodeposited rare earth element alloy films exhibit significant potential as advanced magnetic materials and are promising candidates for specialized applications in aerospace and defense technologies, the automotive sector, as well as medical and advanced electronic devices. Such alloys are characterized by high Curie temperatures, large anisotropy fields, and relatively high saturation magnetization [102]. Amorphous alloys containing iron-group metals are generally ferromagnetic and belong to the class of soft magnetic materials. Their amorphous structure consists of a random atomic arrangement with only short-range order, which leads to randomly distributed local anisotropy directions. Moreover, amorphous films can serve as precursors for the fabrication of nanocrystalline structures upon heat treatment, a process accompanied by the formation of specific intermetallic compounds that can (theoretically) exhibit permanent magnet properties (Table 6).

Among the most well-known are cobalt–samarium intermetallic compounds, such as Co_5_Sm and Co_17_Sm_2_, which show high energy products (120–160 kJ/m^3^ and 159–264 kJ/m^3^, respectively) and excellent thermal stability [103]. These properties enable operation at elevated temperatures in the range of 250–500 °C, owing to their exceptional resistance to demagnetization and high Curie temperatures of 700–800 °C. Sato et al. [62] demonstrated that as-deposited amorphous Co–Sm alloys (from molecular organic bath) with high cobalt contents exhibit high saturation magnetization, while the coercive force increases upon heat treatment. However, both properties remained lower than those expected for bulk materials of the same composition. This was attributed to the fact that the films were not purely metallic due to the incorporation of metal oxides. Similarly, Long [50] reported that an amorphous, samarium-rich film (43 at% Sm, deposited from an aqueous bath) transformed into the Co_17_Sm_2_ phase after heat treatment. Although the magnetic properties improved, they remained inferior to those of the corresponding bulk material, which was attributed to the very small crystallite size and the resulting strong superparamagnetic behavior. Panzeri et al. [95] demonstrated that amorphous Co–Sm deposits of identical composition (20 wt% Sm) exhibited magnetic properties, specifically the in-plane coercivity (H_c_‖), that depended on the presence of glycine in the deep eutectic solvent during electrodeposition. These differences were attributed to variations in deposit morphology, as the glycine-containing bath produced smoother films with a higher degree of crystalline ordering. In contrast, the out-of-plane direction (H_c_⟂) corresponded to the hard axis of magnetization. In contrast, Wei et al. [49] showed that the in-plane coercivity remained nearly constant (about 100 Oe) regardless of the samarium content in the deposits, whereas the coercivity in the perpendicular direction decreased from 800 to 600 Oe as the samarium content in the Co–Sm alloy (deposited from aqueous bath) increased from 1 to 32 at%. In turn, Liu et al. [93] investigated the effect of temperature (−268 °C and 20 °C) on the magnetic properties of as-plated and heat-treated Co–Sm alloys with two compositions (7.9 and 79 wt% Sm). They observed higher coercive fields at lower measurement temperature, while high samarium content significantly improved the magnetic properties. These properties decreased after heat treatment, and no explanation for this behavior was provided. The coercive magnetic field strength at ambient temperature increased slightly from 170 to 175 Oe with increasing samarium content and further to 180 Oe after heat treatment of the high-samarium alloy.

Binary [65,67,71,90,91,92] alloys other than Co–Sm have not been as extensively studied in terms of their magnetic properties. Yuan et al. [71] reported the temperature-dependent magnetic behavior of an amorphous Co–Tm alloy (deposited from a DMSO bath), showing high magnetic performance at −268 °C and soft magnetic characteristics at room temperature. Zhan and Wang [91] demonstrated that the magnetic performance of Co–Gd films depends on both gadolinium content and heat treatment temperature. The amorphous deposits with the highest Gd concentration (55%) exhibited the lowest saturation magnetization and coercive force. Heat treatments in the range of 200–600 °C resulted in different conditions for achieving maximal magnetic properties, with the saturation magnetization reaching a maximum for 600 °C and the coercive force peaking for 400 °C. Notably, heat treatment at 200 °C deteriorated the magnetic properties compared to the as-plated films, reflecting the structure-sensitive nature of the coercive force. Li et al. [90] found that, for as-deposited Fe–Sm, Co–Gd and Ni–La alloys, the coercive field measured perpendicular to the plane at room temperature was greater than that measured parallel to the plane, indicating a clear magnetic anisotropy with the easy magnetization direction lying in the plane of the film. The magnetic anisotropy in these films was attributed to the combined contributions of several factors, including spin–orbit coupling and internal stress.

Some ternary alloy films [53,56,74,77] have been examined for magnetic applications. Gandhi et al. [56] showed that the incorporation of samarium into the Ni–Fe system gradually alters the static magnetization due to an increase in the anisotropy field, requiring higher fields to reach saturation. In turn, Li et al. [74] observed unusual magnetic behavior at −173 °C for a nanograined Co–Dy–Bi alloy under an applied field of 100 Oe, resembling spin-glass behavior, with a freezing temperature of approximately −240 °C.

### 4.2. Electrocatalytic Activity

The electrocatalytic properties of thin alloy films have been investigated for enhanced hydrogen evolution in alkaline aqueous solutions. Studies have shown that the incorporation of lanthanides improves these properties (Table 7) compared to pure iron-group metals such as cobalt [92] or nickel [85,97,98]. The resulting amorphous alloy structure exhibits greater structural disorder and higher electrical conductivity, providing abundant catalytic sites on the electrode surface and facilitating rapid electron transport during the electrochemical hydrogen gas formation. Furthermore, electronic interactions between alloying elements can alter the original electronic structure of the active sites, potentially modifying the adsorption of H species on the catalyst surface and the desorption of adsorbed H_2_, thereby promoting electrocatalytic activity. Although the electrocatalytic performance of these alloys does not surpass that of platinum catalysts or is comparable to alloy films prepared by other methods [85,97], the cost of such catalytic layers is significantly lower.

### 4.3. Microstructure, Microhardness and Corrosion Resistance

The presence of lanthanide ions in electrolytic baths affects the microstructure of the base alloy metal by refining the grain size and improving the surface compactness of the coatings. For example, Wang et al. [59] showed that increasing the LaCl_3_ concentration in nickel electrolytic baths from 0 to 1.2 g/L gradually shifted the corrosion potential of nickel deposits from −0.83 V to −0.74 V, with a simultaneous decrease in corrosion current density from about 1.6 μA/cm^2^ to 0.56 μA/cm^2^ (the La content in the nickel layer was not reported). At the same time, an increase in coating microhardness from 217 HV to 384 HV was observed. A similar trend was reported by Chen et al. [60] for nickel coatings deposited in the presence of lanthanum or cerium ions. They showed a decrease in grain size from 920 nm to 566 nm for CeCl_3_ addition and to 335 nm for LaCl_3_ addition when direct current electrolysis was used. Further grain refinement (532 nm for CeCl_3_ and 126 nm for LaCl_3_) was observed when pulsed current was applied. This corresponded to an increase in coating hardness from 230 HV for pure nickel to up to 320 HV for coatings deposited in the presence of La^3+^ ions under pulsed current conditions. Corrosion resistance was improved with passivation dominating for pulsed current electrodeposited nickel, beneficial in reducing corrosion penetration. A corrosion potential of about −0.2 V and a corrosion current density of 0.5 μA/cm^2^ were reported for pulsed current nickel deposited in the presence of lanthanum salt, corresponding to better properties than those of nickel produced under other deposition conditions and bath formulations.

Li et al. [100,101] investigated the corrosion resistance of ternary Ni–Pr–Mg and Co–Ni–Pr alloys. They showed that the corrosion resistance of Ni–Pr–Mg coatings was better in NaOH solution than in NaCl, as the corrosion potential shifted toward more negative values, below −0.7 V in NaOH compared with values below −0.3 V in NaCl. In both cases, however, the corrosion current densities were comparable, at approximately 1 A/cm^2^ [101]. For Co–Ni–Pr coatings, corrosion tests in NaOH showed variable corrosion potentials, while the corrosion current densities were on the order of 0.001–0.05 A/cm^2^ [100].

## 5. Discussion

Although hundreds of publications are indexed under the topic of electrodeposition of rare earth metals, only a small subset specifically addresses the fabrication of their alloy films. The co-electroreduction of lanthanides has been the primary focus of investigation, despite being challenging due to their low reduction potentials in various electrolyte systems, as the type of electrolyte plays a crucial role in suppressing concurrent hydrogen evolution reactions. Clear trends can be observed in the development of different solution formulations: beginning with aqueous solutions, followed by molecular solvents (which saw the greatest progress in the 1980s–1990s), then ionic liquids from the 2000s, and more recently, deep eutectic solvents. Each class of electrolyte has its own characteristics, advantages, and limitations (Table 8), yet substantial research opportunities remain. This potential arises not only from the diverse possibilities in electrolyte composition but also from different operational modes, especially the largely untapped prospective of pulsed or cycling electroplating techniques.

Most studies on alloy deposition focus on the fundamental characteristics of the electrodeposition process, which is crucial for the development of electrochemistry for rare earth alloys, particularly in novel organic systems. However, challenges remain in conclusively confirming the mechanism of rare earth element electroreduction and clarifying their metallic state within alloys. This is directly related to the suppression of the competing hydrogen evolution reaction, which affects not only the actual deposition rate but also the incorporation of oxide-type species into the growing metallic layer, thereby influencing its properties. To date, no studies have identified a clear solution to this problem. Potentially, water-free, organic-based electrolytes (e.g., Ils and DESs) could be adopted. However, their commercialization and scaled-up demonstrations have not appeared, and the studies remain limited to laboratory achievements and tests. The main reasons for this likely arise from the potentially high costs associated with the following: (i) the need for specialized equipment to prevent electrolyte moisture uptake, (ii) electrolyte preparation, (iii) unfavorable properties of the electrolytes such as high viscosity and lower conductivity compared to aqueous electrolytes, (iv) ensuring a continuous supply of electrolytes from potential manufacturers, (v) unidentified methods for regenerating spent electrolytes, (vi) unknown methods for recycling organic chemicals, (vii) undefined energy requirements, and (viii) corrosion issues in equipment exposed to these new types of electrolytes.

Despite the fact that electrolysis is often described as a “green and cheap method”, the potential industrial implementation of electrodeposition is not straightforward. While no additional chemicals are required for the formation of metallic coatings, as they are produced through the electrochemical reduction of metal ions under the flow of electric current, the codeposition of rare earth element alloys is characterized by low current efficiency, which significantly affects the overall cost. Moreover, the literature lacks data on the electrolysis cell voltage, which is necessary for estimating the actual energy consumption of the process. It is well known, however, that electrolysis is among the most energy-intensive hydrometallurgical processes. Therefore, an economic assessment of the feasibility of this method is essential in the context of the potential benefits in applications of thin alloy films.

The electrodeposition of rare earth metal-containing alloys also faces challenges from competition with other deposition techniques (such as physical and chemical vacuum methods [11]), which can more easily control thin film formation and composition without the barriers posed by side reactions and difficulties in controlling alloy composition due to the specific characteristics of electrolytic baths. An important direction for future research should also include the mechanical properties of thin metallic films. This particularly concerns the role of the substrate in coating adhesion, as well as the prevention of delamination and deformation during usage.

The availability of rare earth elements on global markets cannot be overlooked [104], as it constitutes a fundamental prerequisite for both discussing and fabricating rare earth metal-based materials.

All of these factors constitute significant barriers to scaling laboratory work to industrial production. Undoubtedly, the potential application of electroplated thin films in advanced, miniaturized devices requiring magnetic performance holds the greatest promise and serves as the primary driving force for further advancing both knowledge and practice in this field.

## 6. Conclusions

The analysis of literature data on the electrodeposition of thin films containing rare earth elements allows the following conclusions to be drawn:

1. The selection of electrolyte type (aqueous, organic) and deposition technique plays a decisive role in the incorporation of rare earth elements as metals into the cathodic deposits.
2. The incorporation of oxide-type species during deposition, relatively low current efficiencies, no data on the durability of thin layers, and the lack of information on the stability and regeneration of electrolytes are substantial barriers to the practical implementation of electrodeposition.
3. Further research should focus on clarifying the fundamental mechanisms of rare earth element incorporation, determining the conditions for metal phase formation, providing data on mechanical stability, and evaluating the economic viability of electrodeposition processes.

## Figures and Tables

**Table 1:** Standard potentials (in V vs. SHE) for the electrochemical series of rare earth elements. Data taken from [20].

Pair	Sc	Y	La	Ce	Pr	Nd	Pm	Sm	Eu	Gd	Tb	Dy	Ho	Er	Tm	Yb	Lu
M/M^3+^	−2.08	−2.37	−2.38	−2.34	−2.35	−2.32	−2.30	−2.30	−1.99	−2.28	−2.28	−2.30	−2.33	−2.33	−2.32	−2.19	−2.28
M/M^2+^					−2.00	−2.10	−2.20	−2.68	−2.81			−2.20	−2.10	−2.00	−2.24	−2.76	
M^3+^/M^2+^					−3.10	−2.70	−2.60	−1.55	−0.36			−2.60	−2.80	−3.00	−2.20	−1.05	
M^4+^/M^3+^				+1.72	+3.20												

**Table 2:** Electrodeposition conditions of alloy films from aqueous baths.

Alloy	Bath Type	Current/Potential Conditions *	Deposit Composition	Alloy Properties	Ref.
Fe–Tb	sulfate–citrate, pH 4	Hull cell	8–30 wt% Tb	–	[27]
Co–Tb	sulfate–chloride, pH 1.2–3	PD: −0.8V to −3.5V	50–80 wt% Tb	–	[48]
Co–Gd	sulfamate–glycine, pH 6.5–7	DC: 1–4 A/dm^2^	5 at% Gd	–	[28]
Ni–Nd		DC: 0.2–3 A/dm^2^	5–35 at% Nd	–	
Ni–Sm		DC: 0.2–2.2 A/dm^2^	3–5 at% Sm	–	
Co–Ce	chloride–glycine, pH 6.5–7	DC: 0.2–6 A/dm^2^	2–38 at% Ce	–	
Co–Sm	sulfate–glycine, pH 4	DC: 0.2–7 A/dm^2^, Hull cell	4–24 at% Sm	magnetic	[29,49]
		PC: 0.2–7 A/dm^2^	3–25 at% Sm	–	
Co–Sm	sulfate–glycine, pH 2.5	PD: −1.6V to −2.1V	5–43 at% Sm	magnetic	[50]
Fe–Sm	chloride–glycine, pH 3	DC: 27 A/dm^2^	3–4.5 at% Sm	magnetic	[51]
Ni–Ce–P	chloride–citrate, pH 2	DC: 2.5 A/dm^2^	34 wt% Ce	glass transition temperature	[52]
Ni–Nd–P			34 wt% Nd		
Fe–Tb–Dy	chloride–sulfate–tartrate –citrate	PD: −0.92V to −0.95V	20–60 at% Tb+Dy	magnetic, magnetostriction	[53]
NiCoFe–Tb	sulfate, pH 2.5–3	DC: 2 A/dm^2^	0.8–1% Tb	magnetic, magnetostriction	[54]
NiCoFe–Dy			0.1–0.4% Dy		
NiCoFe–Tb–Dy			1% Tb, 0.5% Dy		
Ni–Ce	chloride–sulfate–citrate, pH 4	DC: 3 A/dm^2^	9 wt% Ce	hydrogen evolution catalyst	[55]
Ni–Ce–Pr			12 wt% Ce, 0.7 wt% Pr		
Ni–Ce–Ho			8 wt% Ce, 1.2 wt% Ho		
Ni–Pr–Ho			0.3 wt% Pr, 0.6 wt% Ho		
Ni–Ce–Pr–Ho			10 wt% Ce, 1 wt% Pr, 1 wt% Ho		
Ni–Fe–Sm	sulfate	DC: 1 A/dm^2^	10–25 at% Sm	magnetic	[56]

* PD—potentiostatic deposition; DC—direct current (galvanostatic) deposition, PC—pulsed current deposition.

**Table 3:** Electrodeposition conditions of alloy films from molecular organic solvents.

Alloy	Bath Type	Current/Potential Conditions *	Deposit Composition	Alloy Properties	Ref.
Bi–Yb	nitrate–chloride–DMSO	PD: −1.6 V	23 wt% Yb	–	[66]
Co–Ce	chloride–sulfamate–DMSO	PD: −1.6 V to −2.8 V	12–36 wt% Ce	magnetic	[67]
		CP: −1.6 V to −2.8 V, 30 mV/s	20–36 wt% Ce	–	
Co–Eu	chloride–toluenesulfonate–DMF	PD: −1.1 V	31 at% Eu	–	[68]
Co–Gd	chloride–toluenesulfonate–DMF	PD: −0.9 V	16–61 at% Gd	–	[69]
Co–Sm	chloride–FM	DC: 1–6 A/dm^2^	20–90 at% Sm	magnetic	[62]
Co–Tm	chloride–nitrate–DMSO	CP: −0.7 V to −2 V, 2–20 mV/s	2–13 wt% Tm	–	[70]
		PD: −2 V to −2.4 V	32 wt% Tm	magnetic	[71]
Fe–Dy	chloride–DMF	PC: 0.25–2.5 A/dm^2^	70 at% Dy	–	[63]
Ni–La	chloride–nitrate–DMF	DC: 0.6 A/dm^2^	18 wt% La		[72]
Ni–Lu	chloride–DMSO	PD: −1.1 V to −2.8 V	10–29 at% Lu	–	[64]
Co–Ce–Mg	chloride–sulfamate–chlorate– DMSO	CP: −1.6 V to −2.6 V, 10–100 mV/s	15–31 wt% Ce, 0–4 wt% Mg	–	[73]
Co–Dy–Bi	chloride–nitrate–DMSO	CP: −0.8 V to −1.2 V, 100 mV/s	12–25 wt% Dy, 45–50 wt% Bi	magnetic	[74]
Co–Er–Bi	chloride–nitrate–DMSO	CP: −0.5 V to −2.8 V, 10–90 mV/s	16–33 wt% Er	–	[75]
Co–Lu–Bi	chloride–nitrate–DMSO	PD: −2.1 V to −3.2 V	10–28 wt% Lu	–	[76]
Co–La–Ni	chloride–chlorate–DMSO	PC: 33 A/dm^2^	0.7–3.9 wt% La, 56–74 wt% Ni	magnetic	[77]
Ni–Lu–Bi	chloride–nitrate–DMSO	CP: −1 V to −2.6 V, 10–85 mV/s	11–23 wt% Lu	–	[78]

* PD—potentiostatic deposition; CP—cyclic (potentiodynamic) deposition; DC—direct current (galvanostatic) deposition; PC—pulsed current deposition.

**Table 4:** Electrodeposition conditions of alloy films from ionic liquid solvents (abbreviation explanation at the end of the article).

Alloy	Bath Type	Current/Potential Conditions *	Deposit Composition	Alloy Properties	Ref.
Bi–Pr	triflate–nitrate–[BmPyr][TFSI]	PD: −2.6 V	29 wt% Pr	–	[80]
Co–Sm	triflimidate–[BmPyr][TFSI]	PP: −0.8/−2 V	21/41 at% Sm	–	[81]
	chloride–triflate–[BmPyr][DCA]	PD: −1.4 to −1.6 V	no data	–	[82]
Fe–Nd	chloride–triflate–[BmPyr][DCA]	PD: −1.6 to −1.7 V	no data		[83]
	chloride–[EMIM][DCA]	PC: 0.4–3.2 A/dm^2^	9–16 at% Nd	–	[84]
Ni–La	chloride–[BmPyr][DCA]	PD: −1.3 V	no data	hydrogen evolution catalyst	[85]
	chloride–[EMIM][Cl]	DC: 0.3–1 A/dm^2^	0.1–9 at% La	–	[86]
Dy–Tb	chloride–[EMIM][BF_4_]	CV: 1.4–2 V	no data	–	[87]
Fe–Nd–B	chloride–triflate–[DMI]	PD: −3.5 V	22% Nd, 8% B	–	[88]
Pt–Y	chloride–nitrate–[N122,201][BF_4_]	PD	no deposit	–	[89]

* PD—potentiostatic deposition; DC—direct current (galvanostatic) deposition; PC—pulsed current deposition; PP—pulsed potential deposition; CV—constant voltage deposition.

**Table 5:** Electrodeposition conditions of alloy films from deep eutectic solvent baths.

Alloy	Bath Type *	Current/Potential Conditions **	Deposit Composition	Alloy Properties	Ref.
Co–Gd	chloride–U–AT–NaBr–KBr	PD: −0.9 to −1.35 V	4–35 at% Gd	magnetic	[90]
	chloride–U–AT–NaBr	DC: 0.5–1.5 A/dm^2^	2.2–55 at% Gd	magnetic	[91]
Co–La	sulfate–chloride–U–NaBr	DC: 0.5–4 A/dm^2^	3–40 at% La	magnetic, hydrogen evolution catalyst	[92]
Co–Sm	chloride–U–AT–NaBr	PD: −1.15 to −1.35 V	2–48 wt% Sm	magnetic	[93]
	chloride–nitrate–ChCl–U	PD: −1.6 to −1.9 V	27–75 wt% Sm	–	[94]
	chloride–ChCl–EG	PD: −0.7 to −0.95 V	0.5–44 wt% Sm	magnetic	[95]
Fe–Nd	chloride–CaCl_2_·6H_2_O–EG	PD: −1.5 V	0.5–4.2 wt% Nd	–	[96]
Fe–Sm	chloride–U–AT–NaBr–KBr	PD: −0.9 to −1.3 V	5–56 at% Sm	magnetic	[90]
Ni–La	chloride–U–AT–NaBr–KBr	PD: −0.9 to −1.35 V	2–20 at% La	magnetic	[90]
	chloride–ChCl–EG	PD: −1.2 V	40% La	hydrogen evolution catalyst	[97]
Ni–Sm	chloride–ChCl–EG	DC: 0.1–0.5 A/dm^2^	2–6 at% Sm	hydrogen evolution catalyst	[98]
Zn–Ce	chloride–ChCl–U	DC: 0.01 A/dm^2^	3 at% Ce	–	[99]
Co–Pr–Mg	chloride–ChCl–U	PD: −0.98 to −1.15 V	4–13 wt% Pr, 2–7 wt% Mg	corrosion	[100]
Ni–Pr–Mg	chloride–ChCl–U	PD: −1 to −1.15 V	6–14 wt% Pr	corrosion	[101]

* U—urea; AT—acetamide; ChCl—choline chloride; EG—ethylene glycol. ** PD—potentiostatic deposition; DC—direct current (galvanostatic) deposition; PC—pulsed current deposition; PP—pulsed potential deposition; CV—constant voltage deposition.

**Table 6:** Comparison of magnetic properties of electrodeposited alloy films (H_c_—coercive magnetic field strength, coercivity, 1 Oe = 0.0796 kA/m; M_s_—saturation magnetization).

Alloy	Alloy Properties	Phase *	Alloy Properties *	Ref.
Co–Gd	H_c_‖: 23.7 kA/m for 55% Gd M_s_: 171 kA/m for 55% Gd	Co_5_Gd	H_c_‖: 35 kA/m for 55% Gd (after heat treatment at 400 °C) M_s_: 550 kA/m for 55% Gd (after heat treatment at 600 °C)	[91]
Co–Sm	H_c_: 2.8–23 Oe for 17–0.5 at% Sm M_s_: 180–2960 emu/cc for 17–0.4 at% Sm	Co_5_Sm, Co_17_Sm_2_	H_c_: 262–81 Oe for 17–5 at% Sm M_s_: 79–620 emu/cc for 17–5 at% Sm	[62]
	H_c_ ⟂: 9.55 kA/m; H_c_ ‖: 6.85 kA/m for 43 at% Sm	Co_17_Sm_2_	H_c_ ⟂: 37.82 kA/m; H_c_ ‖: 32.96 kA/m	[50]
	H_c_ ‖: 270 Oe for 20 wt% Sm (glycine-free bath) H_c_ ‖: 100 Oe for 20 wt% Sm (glycine bath)	no data	no data	[95]
	H_c_: 580 Oe for 7.9 wt% Sm at −268 °C H_c_: 170 Oe for 7.9 wt% Sm at 20 °C H_c_: 2300 Oe for 79 wt% Sm at −268 °C H_c_: 175 Oe for 79 wt% Sm at 20 °C	Co_17_Sm_2_	H_c_: 280 Oe for 79 wt% Sm at −268 °C H_c_: 180 Oe for 79 wt% Sm at 20 °C	[93]
Co–Tm	H_c_: 809 Oe for 32 wt% Tm at −268 °C M_s_: 58.7 kA/m for 32 wt% Tm at −268 °C H_c_: 48 Oe for 32 wt% Tm at 20 °C M_s_: 54.8 kA/m for 32 wt% Tm at 20 °C	no data	no data	[71]
Ni–Fe–Sm	H_c_: 5.3–7 kA/m for 10–25 at% Sm	no data	no data	[56]
Ni–Co–La	H_c_: 214 Oe for 1.25 wt% La M_s_: 23.5 emu/g for 1.25 wt% La	no data	no data	[77]

* After heat treatment.

**Table 7:** Comparison of properties of electrodeposited alloy films as hydrogen evolution catalysts.

Film	Test Solution	Tafel Slope, mV/dec	Exchange Current Density, mA/cm^2^	Overpotential at 10 mA/cm^2^, mV	Ref.
Co	10% KOH	214	0.018	–	[92]
Co–La		192	0.466	–	
Ni	1M KOH	96.2	–	–	[85]
Ni–La		75.6	0.115	190	
Ni	1M KOH	129	0.010	390	[97]
Ni–La		68	0.015	190	
Ni	1M KOH	–	0.21	280	[98]
Ni–Sm		–	3.2	75	
Ni–Ce–Pr–Ho	1M KOH	121.6	–	78	[55]

**Table 8:** Comparison of electrolytes used for electrodeposition of rare earth element alloy films.

Aspect	Aqueous Solutions	Molecular Liquids	Ionic Liquids	Deep Eutectic Solvents
Solvent Type	Inorganic	Organic	Organic	Organic
Pure REE Deposition	No	Yes/No ^1^	Yes/No ^1^	Yes/No ^1^
Alloy Deposition	Yes ^2^	Yes ^2^	Yes ^2^	Yes ^2^
Alloy Structure	Amorphous	Amorphous	Amorphous to crystalline	Amorphous
REE at% in Alloy	Low to medium	Medium to high	Low to medium	Low to medium
Oxygen in Deposit	Yes	Yes	Yes	Yes
Typical Temperature	25 °C	25–35 °C	60–110 °C	70–80 °C
Current Mode ^4^	DC, PC	DC, PC	DC, PC	DC
Potential Mode ^4^	PD	PD, CP	PD, PP	PD
Current efficiency	Low	No data	Medium	No data
Advantages	easy to handle, low cost, high conductivity	wide EW ^3^, medium cost	wide EW ^3^	wide EW ^3^, low toxicity, medium cost
Disadvantages	hydroxide incorporation	moisture-sensitive, volatile solvent	moisture-sensitive, high costs	moisture-sensitive

^1^ Depends on REE. ^2^ Mostly with iron-group metals. ^3^ EW—electrochemical window; ^4^ DC—direct current (galvanostatic) deposition; PC—pulsed current deposition; PD—potentiostatic deposition; CP—cyclic potential deposition; PP—pulsed potential deposition.

## Data Availability

The original contributions presented in the study are included in the article, further inquiries can be directed to the corresponding author.

## Abbreviations

The following abbreviations are used in this manuscript:

[N1114][TFSI]	Butyltrimethylammonium bis(trifluoromethylsulfonyl)imide
[P2225][TFSI]	Pentyltriethylphosphonium bis(trifluoromethylsulfonyl)imide
[N122,201][BF_4_]	N,N-diethyl-N-dimethyl-N-(2-methoxyethyl)ammonium tetrafluoroborate
[BMIM][Cl]	1-butyl-3-methylimidazolium chloride
[EMIM][Cl]	1-ethyl-3-methylimidazolium chloride
[EMIM][BF_4_]	1-ethyl-3-methylimidazolium tetrafluoroborate
[BmPyr][DCA]	1-butyl-1-methylpyrrolidinium dicyanamide
[EMIM][DCA]	1-ethyl-3-methylimidazolium dicyanamide
[BMIM][TFSI]	1-butyl-3-methylimidazolium bis(trifluoromethylsulfonyl)imide
[BmPyr][TFSI]	1-butyl-1-methylpyrrolidinium bis(trifluoromethylsulfonyl)imide
[HMIM][TFSI]	1-hexyl-3-methylimidazolium bis(trifluoromethylsulfonyl)imide
[DMI]	1,3-dimethyl-2-imidazolidione
